# The efficacy of an experimental single solution versus alternate use of multiple irrigants on root dentin microhardness

**DOI:** 10.4317/jced.51007

**Published:** 2013-04-01

**Authors:** Ilgin Akcay, Necdet Erdilek, Bilge H. Sen

**Affiliations:** 1 DDS, PhD, Assistant Professor, Department of Endodontology, School of Dentistry, Ege University, Izmir, Turkey; 2 DDS, PhD, Professor, Department of Endodontology, School of Dentistry, Ege University, Izmir, Turkey

## Abstract

Objectives: This study was carried-out to evaluate and compare the efficacy of various irrigants when used singly or in combination on the microhardness of root canal dentin. Study Design: A total of 50 root-halves were randomly divided into 5 groups immediately after the initial baseline microhardness measurements and treated with: Group-1; 7.5%Ethylenediaminetetraaceticacid (EDTA) + 2.5%sodium hypochlorite (NaOCl), Group-2; 7.5%ethyleneglycol-bis[b-aminoethylether]-N,N,N0,N0-tetraaceticacid (EGTA) + 2.5%NaOCl, Group-3; 7.5%trans1,2diaminocyclohexane NNN’,N’tetraaceticacid (CDTA) + 2.5% NaOCl, Group-4; 7.5%EDTA + 2.5% Ethylenediamine (EDA), and Group-5; 1/1 (v/v) EDTA-EDA mixture + 1/1 (v/v) EDTA-EDA mixture. Fifty mL of each solution was used for 1 minute. The reference and post-treatment microhardness values were measured with a Vickers indenter under 80-gram load, 15-second dwell time. Data were analyzed by two-way ANOVA and Bonferroni tests (p=0.05). Results: All solutions decreased microhardness of dentin (p< 0.05). There was statistically significant difference between each group, except Group-1 and 4, after 1st solution application. While Group-2 resulted in a greater reduction of dentin hardness, Group-5 caused the least change in microhardness values, after 1st solution application (p< 0.05). No statistical difference was observed between Groups 1-4, after 2nd solution application. However, Group-5 showed a significant difference compared with all other groups, after 2nd solution application (p< 0.05). Conclusions: Under the experimental conditions, all tested solutions reduced the microhardness of the root canal dentin. EGTA was the most efficient chelating agent. EDTA-EDA single mixture has led to least change on the microhardness of root dentin.

** Key words:**Microhardness, CDTA, EDTA, EGTA, Ethylenediamine.

## Introduction

It is important to emphasize that chemical and mechanical procedures of root canal instrumentation cannot be separated in any manner, because the objective is to obtain the trinomial: cleaning, shaping and disinfection of root canal. However, during irrigation, chemicals that are used cause alterations in the chemical composition of root dentin, which in turn may decrease microhardness (1). During irrigation, the hard tissues of a tooth are exposed to solutions deposited in the pulp chamber. This action facilitates the instrumentation, howbeit it may cause alterations on dentin and enamel surface. When it becomes significant, it can affect the resin adhesion and sealing ability of the sealers to the root dentine walls (2,3). It has also been reported that adhesive systems bond significantly better to calcified dentin than to decalcified dentin (3); therefore, it is significant to test the effect of the irrigation solutions on dentin microhardness.

Chelating agents were included in endodontic practice by Nygaard-Østby (4). The liquid form of EDTA was used to chemically soften the root canal dentin, to dissolve the smear layer and to increase dentin permeability (5). Studies have shown that different concentrations of EDTA, EGTA and CDTA have demineralizing capacity and are capable of decreasing the microhardness of root canal dentin (2,6-10). The decalcifying effect of chelating agents depends largely on application time, solution pH, and concentrations (11).

Sodium hypochlorite has been the irrigant of choice in endodontic practice because of its antimicrobial action and capability to dissolve organic tissues (12). Although EDTA is the most well-known and used chelating agent in endodontic therapy, neither of these solutions could remove the smear layer completely (13). Ideally, a single root canal irrigant should be able to debride the root canals, remove smear layer and provide antimicrobial property. However, a solution like this, still not exists. Therefore, the synergetic effect between NaOCl and the chelating agents’ acting on the smear layer is of necessity for biomechanical preparation (4,14,15). The alteration in dentin microhardness has also been shown due to the NaOCl use (16-18).

Ethylenediamine (1,2, Diaminoethane) is a hygroscopic liquid with an ammoniacal odour. Although EDA finds its chief application in the chemical industry as an intermediate in the synthesis of organic compounds, it is also used to some extent as a solvent for water-insoluble acids, resins, and gums (19). EDA can also solubilize albumins, casein and type I, III collagen (20). Aktener and Bilkay have demonstrated that a single mixture of EDTA and EDA was found to be effective in removal of smear layer (13).

The effects of some irrigation solutions, such as EDTA, EGTA, CDTA and NaOCl on dentin hardness have been previously evaluated (6,10). However, the effect of EDA or EDTA-EDA mixture on microhardness of dentin has not been evaluated yet. Therefore, this study was designed to evaluate the effect of EDTA-EDA single mixture or sequential use of chelating agents and organic solvents.

## Material and Methods

- Preparation of samples

Twenty-five human canine teeth, recently extracted for periodontal reasons, with similar dimensions were used. All teeth were stored in 0.01% thymol solution, and the soft tissues covering the root surfaces were removed with gauze and a fine brush. The crowns were removed at the cemento-enamel junction with a high-speed bur under water cooling. The roots were split in the buccolingual direction to obtain 2 root halves. Fifty samples were obtained, and embedded in polyester resin blocks. The dentin surfaces of the mounted specimens were ground smooth using increasingly finer emery papers to remove any surface scratches and were finally polished with alumina suspension (No: 2, 3000 Ǻ, Presi, Grenable, France).

- Determination of microhardness

Dentin microhardness of all root sections was initially measured on a Carl Zeiss Jena M 1192 (Hegestelft, Germany) microhardness tester with a Vickers diamond indenter at X35 magnification and recorded as control values in Vickers number before the soaking process. Each root section was divided into 3 segments as coronal, middle and apical third. Accordingly, 3 separate indentations parallel to the edge of the root canal lumen, at depth of 100 µm from the pulp-dentin interface, each using 80-gram load and 15-second dwell time were made at 3 segments of the root in each sample, taking care to avoid any overlap between them. The length of the two diagonals was used to calculate the microhardness value (Vickers Hardness Number [VHN]). The representative hardness values were obtained as the average of the results for the nine indentations (VHN1).

- Soaking phase

The specimens were treated with the solutions immediately after the initial baseline measurements. The 50 specimens were randomly divided into 5 groups according to the irrigation solution used. The treatment groups and procedures are demonstrated in  Table 1. The application time for all solutions was 1 min. The specimens in each group were soaked in first solution, rinsed in distilled water and blotted dry. After measurement of microhardness (VHN2), the specimens were exposed to second solutions, rinsed again in distilled water and blotted dry. Then, final microhardness measurements were done (VHN3).

**Table 1:**
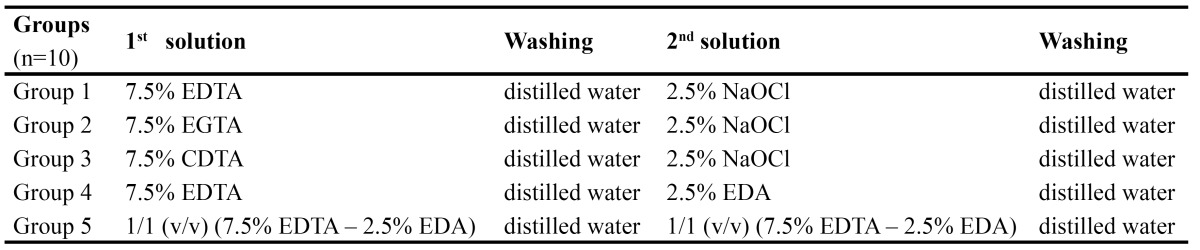
Sequential procedures (solution volume= 50 ml; application time= 1 min).

Data were analyzed statistically using two-way ANOVA. When significance was detected, covariance analysis and Bonferroni tests were used to determine statistical differences among solutions. All hypothesis testing were performed at the 95% level of confidence.

## Results

**Figure 1:**
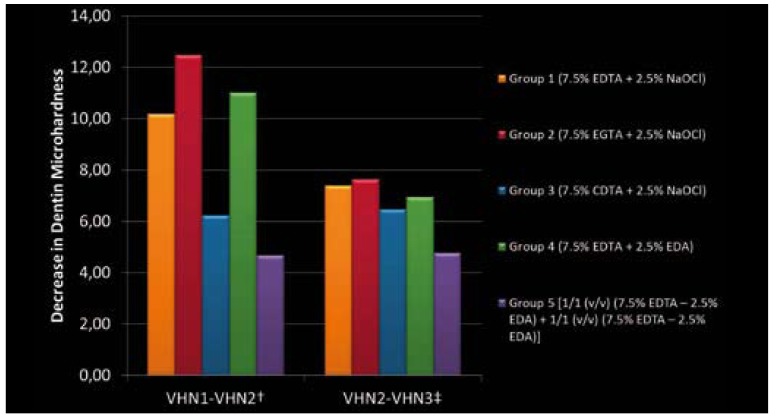
The decrease in microhardness values after 1st and 2nd solution applications.

 Table 2 shows the mean values of decrease in dentin microhardness, after 1st and 2nd applications. The results indicated that all solutions significantly decreased microhardness of root dentin (p < 0.05) (Fig. 1). There was statistically significant difference between each group, except Group 1 and Group 4, after 1st solution applica-tion. According to the results, treatment with 7.5% EGTA (Group 2) resulted in a greater reduction of dentin hardness (p < 0.05). However, treatment with the single mixture of EDTA-EDA (Group 5), caused the least change in microhardness ( Table 2). The results indicated that, sequential use of 2.5% NaOCl with the chelating agents as a final solution caused significant reduction in microhardness values (p < 0.05). Nevertheless, the effect of EDA solution (Group 4) on root canal microhardness was similar with the effect of NaOCl, in Groups 1, 2 and 3 (Fig. 1). It was observed that EDTA-EDA mixture (Group 5) led to minimum decrease on dentin microhardness after 1st and 2nd applications (p < 0.05) (Fig. 1). After the application of the 2nd solutions, there were no significant differences between Groups 1, 2, 3 and 4. However, Group 5 showed a significant difference compared with all other groups, after 2nd solution application.

**Table 2:**
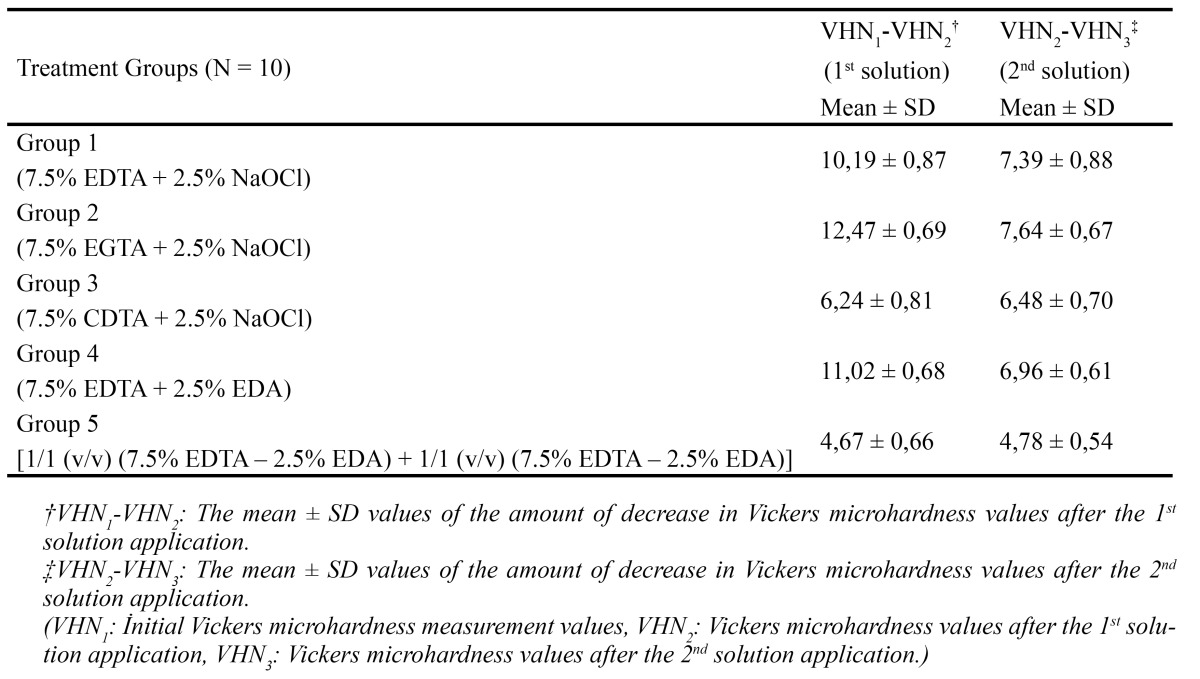
The amount of decrease in root canal dentin microhardness values after 1st and 2nd solution applications.

## Discussion

Little information is available on the direct interaction of EDTA and EDA in the endodontic literature (13). This would appear to be the first report on the efficacy of dentin microhardness of EDTA/EDA combination. In this study, the effect of several chelating agents with subsequent use of organic tissue solvents (NaOCl and EDA) on root canal dentin microhardness was evaluated.

In previous studies, it has been shown that EDTA solution induces a decrease in root dentin microhardness and our results support this conception (1,7,21,22). EDTA binds to calcified components (particularly Ca+2 ions) of dentin through chelating action; thus, in turn, it causes demineralization and softening of dentin (4). This process applies for demineralization mechanism of other chelating solutions such as EGTA and CDTA (2,6,9,10). Although, there is a lot of research in medicine on chelating solutions like EGTA and CDTA to detoxify heavy metals that contaminate patients, only a few studies measured the effect of EGTA and CDTA solutions on root dentin (2,6,9).

CDTA possesses extremely high affinities for both magnesium and calcium, unlike EGTA which has a selec-tively high affinity for calcium only. It was demonstrated that, in the presence of EGTA, magnesium remains uncomplexed by chelator, whereas in the presence of CDTA, more than 99% of the magnesium remains com-plexed by the chelator (23).

Cruz-Filho et al. initially evaluated the effect of 5% EGTA on radicular dentin microhardness for 5 minute and showed that the solution reduced dentin microhardness significantly (2). Although the application time decreased to 1 minute, there was a slight increase in the solutions percentages tested in this study. Herein, 7.5% EGTA and CDTA were used, in terms of impact on the results due to the reduced duration of the application and to not to evaluate concentrations of the solutions as a variable. The results showed that EDTA decreased the microhardness ≈10-11, EGTA ≈12.40 and CDTA ≈6.20 in VHN. Because of the high affinity of EGTA for Ca2+ with regard to EDTA and CDTA, there was significantly higher decrease in dentin microhardness (23).

Seventeen percent EDTA has the potential for causing excessive dentinal erosion if the application time exceeds 1 minute (24). Furthermore, 3-minute 8% EDTA irrigation was reported to be as effective as 1 minute 15% EDTA irrigation (25). Thus in the present study, all tested solutions had application time which was limited to 1 minute.

Dentin is composed of about 50 vol% mineral in the form of a carbonate rich, calcium deficient apatite; 30 vol% organic matter which is largely type I collagen; and about 20 vol% fluid, which is similar to plasma but is poorly characterized (26).

Previous investigations have shown the suitability and feasibility of Vickers microhardness test for evaluating the changes on the surface of dental hard tissues treated with chemical agents (1,7,16). Therefore, Vickers microhardness test was preferred in this study due to the method’s practicality. In this study, microhardness values were measured after each soaking process. The reason for this was to mimic the final irrigation and to see if the solutions affect the microhardness of dentin during the process.

The solubilizing efficacy of EDA on albumins, casein and type I, III collagen is known (20). It was also demon-strated that a single mixture of EDTA and EDA (5%) was found to be effective in removal of smear layer (13). The advantage of this single mixture is that it has chelating affect as well as organic solvent action. It combines the property of dissolution of both organic and inorganic component of smear layer in a single solution (13). (EDTA-EDA) mixture 1/1(v/v) showed the least effect on root canal dentin microhardness. Even plain EDTA, when used, has self-limiting property (27), a self-limiting chemical interaction may have occurred between the solutions, in the mixture. Therefore, the mixture was not as effective as plain EDTA solution, on the hardness of dentin.

There are many studies suggesting the combined use of EDTA with NaOCl (14,15,28). Consequently, com-bined use of organic solvents such as EDA with EDTA may limit the adverse efficiency of these chemicals and may prevent the excessive dissolution of organic and inorganic content of dentin. Thereby, there may be no immoderate decrease in dentin microhardness.

In addition, the organic dissolving properties of NaOCl on the collagen component of dentin explain how the alternated irrigation with these solutions affects the hardness of dentin (29). White et al. reported a 59% reduc-tion in the strength of dentin after exposure with 5.25% NaOCl (30). Slutzky-Goldberg et al. also measured the effect of 2.5% NaOCl irrigation on root dentin and reported a decrease in microhardness after the treatment (17). It was suggested that EDTA might decalcify peritubular dentine during the early stages of root canal irrigation and that the subsequent use of NaOCl dissolves the exposed organic matrix. Due to these complementary effects erosion may occur on the dentinal wall (29). Researchers evaluated the decrease in root dentin microhardness by applying 17% EDTA, 5.25 and 2.5% NaOCl for 15 min, and indicated that all of the solutions decreased the microhardness (16). After treating with 17% EDTA for 2.5 min and 5.25% NaOCl for 2.5 min Eldeniz et al. showed a decrease in the root dentin microhardness, and stated that, it is caused by the demineralization effect of the solutions (1).

In this study, it was observed that the effect of EDA solution on root canal microhardness was close to NaOCl after treatment with EDTA (comparison of Group 1 & 4). The organic dissolving properties of EDA on the collagen component of dentin may explain these results (20). Consequently, it comes to mind that both organic dissolvent solutions affect the collagen structure in similar range. Thereby, it can be speculated that changes in the properties of the collagen may influence the hardness.

The soaking process of (EDTA-EDA) mixture [1/1 (v/v)] was repeated and measured twice, to mimic the final irrigation and to standardize the process applied to the groups. We observed that soaking the samples in 1/1 (v/v) (EDTA-EDA) mixture after treatment with the same mixture had the least effect on dentin microhardness. This may be caused by a self-limiting interaction that has occurred in the mixture.

Under the experimental conditions, all tested solutions reduced the microhardness of the root canal dentin. EGTA was the most efficient chelating agent. EDTA-EDA single mixture has led to least change on the microhardness of root dentin. The present observations suggest that root canal irrigation with different chemical solutions either chelating agent or organic dissolvent leads to structural changes as evidenced by the reduction of dentin microhardness. The efficacy of lower concentrations of EGTA & CDTA and the chemical interaction of EDTA-EDA mixture merits further evaluation. There are also many questions to be answered as to the extent of the efficacy of EDA on organic tissue dissolution and antimicrobial capacity. Nevertheless, using an irrigation solution which is less toxic but as effective as NaOCl can clinically be more reliable.
